# The post-vaccine microevolution of invasive *Streptococcus pneumoniae*

**DOI:** 10.1038/srep14952

**Published:** 2015-10-23

**Authors:** Amelieke J. H. Cremers, Fredrick M. Mobegi, Marien I. de Jonge, Sacha A. F. T. van Hijum, Jacques F. Meis, Peter W. M. Hermans, Gerben Ferwerda, Stephen D. Bentley, Aldert L. Zomer

**Affiliations:** 1 Radboud university medical center, Laboratory of Pediatric Infectious Diseases, Nijmegen, The Netherlands; 2 Radboud university medical center, Bacterial Genomics Group; Center for Molecular and Biomolecular Informatics, Nijmegen, The Netherlands; 3 Canisius-Wilhelmina Hospital, Department of Medical Microbiology and Infectious Diseases, Nijmegen, The Netherlands; 4 Radboud university medical center, Department of Medical Microbiology, Nijmegen, The Netherlands; 5 Wellcome Trust Sanger Institute, Pathogen Genomics group, Hinxton Cambridge, United Kingdom

## Abstract

The 7-valent pneumococcal conjugated vaccine (PCV7) has affected the genetic population of *Streptococcus pneumoniae* in pediatric carriage. Little is known however about pneumococcal population genomics in adult invasive pneumococcal disease (IPD) under vaccine pressure. We sequenced and serotyped 349 strains of *S. pneumoniae* isolated from IPD patients in Nijmegen between 2001 and 2011. Introduction of PCV7 in the Dutch National Immunization Program in 2006 preluded substantial alterations in the IPD population structure caused by serotype replacement. No evidence could be found for vaccine induced capsular switches. We observed that after a temporary bottleneck in gene diversity after the introduction of PCV7, the accessory gene pool re-expanded mainly by genes already circulating pre-PCV7. In the post-vaccine genomic population a number of genes changed frequency, certain genes became overrepresented in vaccine serotypes, while others shifted towards non-vaccine serotypes. Whether these dynamics in the invasive pneumococcal population have truly contributed to invasiveness and manifestations of disease remains to be further elucidated. We suggest the use of whole genome sequencing for surveillance of pneumococcal population dynamics that could give a prospect on the course of disease, facilitating effective prevention and management of IPD.

The bacterial pathogen *Streptococcus pneumoniae* remains an important cause of pneumonia and meningitis worldwide, causing an estimated 1.6 million deaths annually^1^. Immunization with a 7-valent pneumococcal conjugated vaccine (PCV), both in young children^2^ and the elderly^3^, has been demonstrated to be highly efficacious in preventing invasive pneumococcal disease (IPD) caused by vaccine serotypes. Moreover, after the introduction of routine pediatric vaccination, an additional decrease in vaccine-type pneumococcal infections occurred, both in pediatric carriage^4^,^5^ as well as in IPD at all ages^6^, indicative of a herd immunity effect. However, in all regions where routine PCV vaccination has been introduced, we see replacement in pneumococcal carriage and disease by non-vaccine serotypes^7^,^8^,^9^, which at least partially abrogates the preventive effect of vaccination. In the Netherlands, the number of reported cases of IPD has not decreased after vaccination due to full replacement by non-vaccine serotypes^10^.

Currently available pneumococcal vaccines target the pneumococcal polysaccharide capsule. Although over 90 antigenically distinct pneumococcal capsules (denominated as serotypes) are known to date, only a selection of 7 serotypes (later extended to 10 and 13) is included in the PCV, based on modelling vaccination effects by the frequency and the propensity for invasiveness for each serotype^11^,^12^. Whereas the polysaccharide capsule is essential for the pneumococcus to cause IPD^13^, recent publications suggest that true determinants of invasiveness may lay underneath the pneumococcal capsule, represented by variations on the pneumococcal genome^14^,^15^,^16^,^17^,^18^, which should be included in modelling vaccine effects^19^.

Since the introduction of routine pediatric immunization with PCV, several studies have detailed pneumococcal genomic epidemiology in pediatric carriage and otitis media under vaccine pressure. They all demonstrated that the pneumococcal genomic structure of pediatric carriage remained fairly stable, and that serotype replacement occurred mainly through expansion of previously existent clones^20^,^21^,^22^,^23^,^24^,^25^. The single study that examined whole pneumococcal genomes, including virulence factors, reported little effect on the accessory genome at the overall pneumococcal carriage population level despite massive serotype replacement^26^. However, the effect of pneumococcal vaccination on whole genome epidemiology of invasive pneumococcal disease remains unexplored, although it may hold invaluable information on understanding the long term effects of mass vaccination, especially with regard to changes in clinical manifestation of disease due to the changing prevalence of virulence factors in the pneumococcal population.

PCV7 was introduced in the Dutch National Immunization Program in April 2006 while PCV10 was introduced in May 2011. In this study, we have sequenced 349 invasive pneumococcal isolates from 2001 to June 2011, in order to investigate the herd-immunity effect of PCV7 on pneumococcal population genomics in IPD.

## Results

### Cohort and serotype dynamics

We serotyped and sequenced 349 strains of *S. pneumoniae* isolated from an unbiased cohort of IPD patients in Nijmegen, the Netherlands, between 2001 and 2011. In the Netherlands, the elderly are not routinely vaccinated against pneumococcal disease, pediatric pneumococcal vaccination coverage has been high (>94%)^27^, and penicillin resistance was below 3% during the entire study period^28^. These facts increase the probability that in this cohort, major changes in adult IPD epidemiology are due to herd immunity after pediatric immunization. Moreover, risk factors for acquiring a pneumococcal infection have not changed for the IPD patients in our study cohort, suggesting that replacement has been a merely pneumococcus-mediated phenomenon. According to our analysis, since the 7-valent PCV (PCV7) was introduced in the pediatric Dutch National Immunization Program in April 2006, the invasive pneumococcal population structure has altered substantially. Its introduction preluded a steady decrease in adult IPD cases caused by the seven serotypes it protects against (vaccine types, VTs): 4, 6B, 9V, 14, 18C, 19F, and 23F, supporting a herd immunity effect of PCV7, concordant with a previous nationwide study^29^. In addition, the absolute increase in IPD cases caused by non-PCV7 protected serotypes (non-vaccine types, nVTs) observed in the Netherlands^30^, as well as within our cohort^31^ is suggestive of serotype replacement or capsular switching. Similarly to the pediatric pneumococcal carriage study by Croucher *et al.*^26^, in our non-vaccinated IPD cohort we observed an increase of subtype variants of VTs: 6A, 19A, 23A, and 23B. However, the post PCV7 nVT IPD cases were dominated by serotypes 1, 3, 7F, and 8 which may be seen less frequently in carriage cohorts^11^.

### Core genome phylogeny

**Figure 1:**
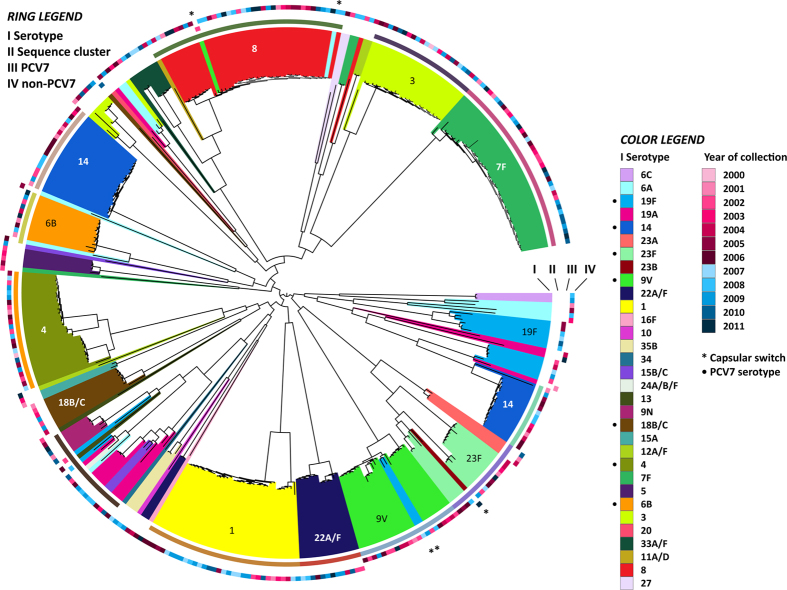
Structure of the invasive pneumococcal population. The maximum likelihood phylogeny was generated using 59,682 polymorphic sites within a 970,559 bp codon alignment of 755 core OGs with similar phylogenetic profiles as ribosomal protein encoding genes in order to exclude genes that were acquired by, for example, horizontal gene transfer. The inner bars each represent a pneumococcal strain (I) and are colored by capsular serotype. The second circle displays the sequence clusters (II). The year of collection is marked by the color (red: pre-PCV7, blue: post-PCV7) and intensity of coloration per year, for PCV7 vaccine serotypes (III) and non-vaccine serotypes (IV).

Phylogeny of the 349 IPD isolates was reconstructed based on a ‘superalignment’ of concatenated alignments of genes in 786 out of the 1075 core orthologous groups (OGs)—containing genes present in a single copy in all isolates. 289 OGs with a dissimilar tree topology and branch distance as compared to ribosomal protein encoding genes were excluded as their phylogeny may be influenced by recombination or homoplasies. A tight clustering of isolates by serotype was observed (Fig 1). Similar to the carriage study by Croucher *et al.*^26^, in our IPD study, serotype replacement was responsible for the increase in nVTs. The rare cases of capsular switch were probably extant before immunization and did not increase upon the introduction of PCV7. It is therefore unlikely that capsular switching was induced by vaccine pressure, although larger numbers of isolates may be needed to confirm this. Compared to their closest neighbors in the phylogenetic tree, capsular switched strains accumulated only between 1 and 10 single nucleotide polymorphisms (SNPs) in their core genome. The majority of these SNPs occurred in regions centered on genes involved in resistance to and efflux of antibiotics, synthesis of cell wall proteins, as well as transposases, pili and phage elements (Supplementary Table 1). A similar situation with regard to gene insertions or deletions was observed in the capsule switched isolates, suggesting phages and transposing elements play a major role in shaping the pneumococcal genome on relatively short timescales (Supplementary Table 2). The sequence clusters were based on BAPS analyses, and were determined to study if serotype replacement within a serogroup would involve closely related core genomes. Although this holds true for serogroup 23, replacement in serogroups 6 and 19 is scattered over the phylogenetic tree.

### Genome diversity

**Figure 2:**
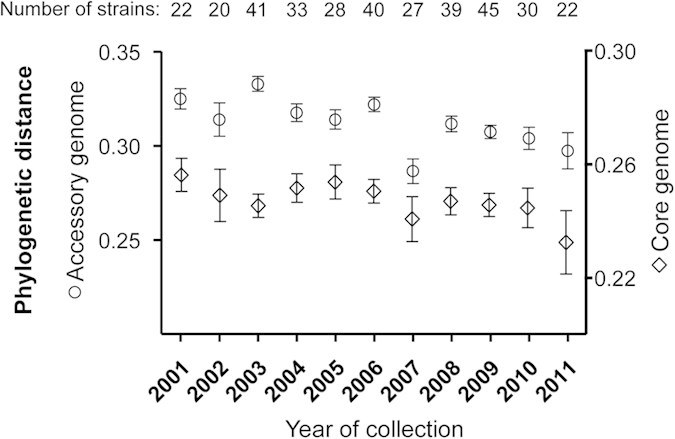
Annual diversity among accessory genomes. The diversity was calculated between strains collected within the same year. Using a binary measure of the presence (1) or absence (0) of a gene in each accessory OG, diversity among strains was calculated by applying the distance measure described by Dutilh *et al.*^47^. Core OG diversity was determined by comparing the pairwise distances between isolates from the phylogenetic tree per year. *Circles: means accessory distance; diamonds: means core OG distance; whiskers: 95% confidence intervals.*

**Figure 3:**
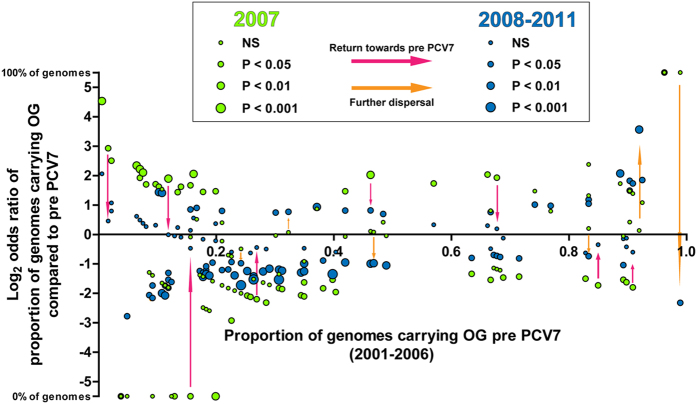
Temporal stability of accessory gene pool. Odds ratios for prevalence of individual OGs in the accessory genome whose frequency significantly altered at bottleneck shortly post PCV7 (2007) or during re-expansion (2008–2011), compared to pre PCV7 (2001–2006) The X-axis indicates the proportion of isolates carrying each OG in 2001 while the Y-axis indicates the log transformed odds ratio of strains carrying each OG in 2007 (green dots), and post PCV7 (blue dots), relative to pre PCV7.

Analysis of the diversity of the accessory genome among strains revealed a significant drop in OG diversity in 2007 shortly after the introduction of PCV7 (p < 0.0001), and a re-expansion in subsequent years (Fig. 2); a similar trend is seen for the core OG diversity, although here the 95% CIs overlap. This illustrates that vaccination had an early temporary bottleneck effect on IPD gene diversity. In concordance with the decreased diversity after vaccination, a considerable number of OGs were significantly altered in prevalence, but most of them returned towards equilibrium afterwards (Fig. 3). Majority of the OGs that contributed to the re-expansion of diversity in the accessory genome after 2007 was comparable to those circulating pre PCV7. The decreased diversity after vaccination when VT stains were steadily removed from the population seems to represent the simultaneous decrease of certain sets of VT-related genes. However, the re-expansion of diversity with similar genes, suggests that those might now be carried by replacing nVT strains. Such a phenomenon has recently been predicted by modeling from pneumococcal carriage genome data^32^. Aside from this tendency to recover, certain OGs dispersed from their prevalence in the original gene pool. Both dynamics will be detailed below. A second gradual decrease in accessory genome diversity was observed towards 2011. This was not due to a distinct decreasing diversity among VT strains post vaccination, as the diversity in the accessory genomes remained similar between VT and nVT strains (see Supplementary Figure 1).

### Gene dynamics

For the entire cohort, 506 pneumococcal genes were significantly overrepresented in the group of VTs, and 223 in nVTs (see Supplementary Table 3). This observation suggests that the distribution of particular genes over strains is more clustered in the VT group. Given the low number of serotypes in the VT group, it may indicate that also the phylogeny of the accessory genome clusters by serotype. Whereas 97 genes were exclusively found in the VTs and 331 genes were unique to the nVTs, the average number of unique genes per serotype was equal.

Post PCV7, 105 genes significantly decreased in frequency, among which many phage-related genes and transposons that went down along with VTs carrying them. Only 33 genes expanded significantly, including mainly ABC-transporters, zinc metallopeptidases and genes facilitating recombination. The fact that VT strains had more OGs associated with phages throughout the course of the study may relate to the ‘kill the winner’ strategy employed by phages as VT strains had a higher prevalence and would therefore be more likely to be a target for phage predation.

Under the selective pressure of herd immunity post PCV7, the rate of adult IPD caused by VTs decreased. Those VT strains that still caused IPD after the PCV7 bottleneck more often carried OG0185 DNA methylase (1.8 fold increase, p = 0.008), OG1539 flippase wzx associated with serogroup 6 (2.3 fold increase, p = 0.037), and a cluster consisting of 13 Tn5253-related genes (3.7 fold increase, p = 0.02). Although the latter are often related to acquisition of antibiotic resistance, all strains concerned were fully susceptible to tetracycline and clindamycin, and only 1 was resistant against erythromycin and clarithromycin. The patients involved had no notable history indicative of increased antibiotic pressure (antibiotics use before admission, comorbidities associated with frequent antibiotics use, nursing home residency). Therefore, presence of the Tn5253 cluster may have facilitated maintenance in IPD through a different mechanism.

After 2007 the number of adult IPD cases caused by non-vaccine serotypes expanded. Among nVT IPD, the proportion of strains carrying OG1150 (toxin antitoxin component HicA) increased post PCV7 (p = 0.032). Genes that were first observed in nVTs after PCV7 (n = 115) were infrequent. They coded for phages, transposons and membrane structures, but also included OG2585 (endonuclease), and OG2354 (multidrug resistance transporter MdtK). Although the latter is homologous to the MepA multidrug transporter of *S. aureus*, here a different antibiotic resistance pattern was observed. Of the two strains carrying MdtK, one isolate was fully susceptible *in vitro*, while the other was resistant to clarithromycin,^33^ intermediately susceptible to doxycycline and ofloxacin, but not resistant to fluoroquinolones - the substrate of MepA in *S. aureus*^31^.

Although capsular switching has been rare, theoretically, individual pneumococcal genes may also benefit from translocation to strains with a polysaccharide capsule not targeted by vaccination. We studied whether individual genes shifted from VT to nVT strains by comparing the odds ratios for linkage to VT versus nVT strains before and after the PCV7 bottleneck. Of the 86 genes that displayed increased odds for being related to nVTs after PCV7, 72% were previously related to VTs, indicative of a certain originality to nVT strains. Whether these genes actually benefit from a transition towards nVT strains, or contribute to severity of infection remains to be elucidated.

Although after the introduction of PCV7 the overall severity of IPD cases (measured by pneumonia severity index) remained stable, higher rates of pleural effusion and empyema have been reported^31^,^34^, which may be attributed to a changed frequency of virulence factors in the pneumococcal population. For instance zinc metalloproteinase C (*zmpC*), mainly present in nVTs, has been associated with a more severe clinical manifestation of IPD and was shown to be expanding^17^. This illustrates that elimination of VTs may result in substitution by nVT strains that potentially confer more severe disease. Modeling of vaccination-induced changes in frequency of disease associated genes may therefore be of interest to optimize target selection in new generations of pneumococcal vaccines. Such an approach would require further exploration of the role of pneumococcal genotypes in the manifestation of human infection.

## Conclusions

Despite serotype replacement in pneumococcal disease, after pediatric pneumococcal vaccination with PCV7 we observed a temporary bottleneck in gene diversity, which re-expanded mainly by genes already present in the original gene pool. Our observations suggest that the introduction of PCV7 has only temporarily affected the pneumococcal population, like disease frequency, with the effects lasting up to one year. Certain genes in IPD have dispersed from their original prevalence, while others became overrepresented in VT, or shifted towards nVT. These changes may influence clinical manifestation of disease if these genes are associated with disease. We suggest whole genome sequencing to investigate pneumococcal dynamics after vaccination and as such maintain close surveillance of strains in a population; information that could give a prospect on an altered course and severity of disease, facilitating effective prevention and management of invasive pneumococcal disease.

## Methods

### Pneumococcal strain collection, DNA isolation and serotyping

Pneumococcal strains were isolated from adults hospitalized with a bacteremic pneumococcal infection at two Dutch hospitals between January 2000 and June 2011 as described by Cremers *et al.*^31^. The candidates were retrospectively included in the Pneumococcal Bacteraemia Collection Nijmegen (PBCN). This observational cohort study was approved by the Local Medical Ethics Committees of both participating hospitals. The cohort was divided in pre and post PCV-7 strains as defined by their collection from blood before or after January 1 2007. The pneumococcal serotypes were determined using the multiplex PCR analysis^35^. In case multiplex PCR was inconclusive, serotyping was complemented with the Quellung reaction using Pneumococcus Neufeld Antisera (Statens Serum Institute, Copenhagen, Denmark) according to the manufacturer’s instructions^31^. All pneumococcal serotypes were then confirmed using an in-house implementation of the Sanger Institute molecular capsular typing (MCT) system^36^,^37^.

### Genomic DNA preparation and whole genome sequencing

The strains were grown statically in 10 ml of Todd Hewitt broth (Merck, Darmstadt, Germany) with 5% yeast extract at 37 °C and 5% CO_2_ to an optical density at 620 nm of 0.2–0.3. The bacterial pellet was washed with 1 ml PBS and DNA was extracted using QIAGEN Genomic-tips 20/G and Genomic DNA Buffer Set (both Qiagen, Venlo, The Netherlands) according to the manufacturer’s instructions for mini DNA preparations. The concentration of extracted genomic DNA was determined using Quant-iT™ PicoGreen® dsDNA Reagent (LifeTechnologies, Bleiswijk, The Netherlands) and the TECAN GENios plate reader with Magellan software (Tecan, Giessen, The Netherlands) and its intactness was confirmed with gel electrophoresis. Genomic DNA was sequenced on an Illumina HiSeq 2000 as paired-end reads of 100 nucleotides.

### Genome assembly, mapping and annotation

The strains were assembled using the Sanger Institute genomes assembly pipeline. For each strain, Velvet^38^ was used to create multiple assemblies by varying the kmer size between 66% and 90% of the read length. From these assemblies, the one with the highest N50 was chosen and contigs that were shorter than the insert size length were removed. The resulting assembly was improved by the following steps: The contigs of the assembly were scaffolded by iteratively running SSPACE using default settings^39^. Then, gaps identified as 1 or more N’s, were targeted for closure by running 120 iterations of GapFiller^40^. Genomes were annotated using an in-house implementation of Prokka^41^. The genome assemblies were deposited at the European Nucleotide Archive under study number ERP001789.

### Gene clustering and phylogenetic analysis

All putative coding sequences were translated and analyzed by an all-versus-all blastP employing a 10E-15 *e-value* cut-off and BLOSUM90 matrix. TribeMCL^42^ was used to cluster orthologs by an inflation value of 4 to implement the MCL step, which resulted into a total of 3021 OGs. Of these OGs, 1075 were denominated as core OGs as they consisted of proteins present in single copies in each of the 349 strains. Protein sequences of these core OGs were aligned with MUSCLE^43^, and subsequently codon translated into nucleotide alignments^44^. Genes of OGs encoding ribosomal proteins from each strain were concatenated into a single ‘ribosomal’ alignment. A reference maximum likelihood phylogeny was generated based on this ribosomal alignment using RAxML^35^. Phylogenies for each of the remaining core OGs were also generated separately. Plotting the Euclidian distances (EuD) of each OG tree with the ribosomal protein encoding tree resulted in three distributions of distances. To reach an alignment with high phylogenetic resolution, the sequences from OGs whose phylogenies were similar to the ribosomal phylogeny (EuD ≤ 0.03) were concatenated to the ribosomal alignment to give a single reduced ‘core’ super-alignment. Finally, polymorphic regions of this super-alignment were extracted and re-analyzed with RAxML using a general time-reversible (GTR) nucleotide substitution model. Core OG diversity was determined by comparing the average of the pairwise distances between the isolates per year. The reduced core super-alignment was also analyzed using BAPS; Bayesian Analysis of Population Structure software^46^ to determine the sequence clusters. Two runs of 40 and 50 maximum clusters were performed, each creating 12 largely monophyletic sequence clusters and an extra ‘sink’ cluster that incorporated all unclassified isolates. Visualization of the tree was performed using iTOL^47^.

### Analysis of the population structure

Core genomes and whole genomes of strains with a capsule switched serotype were separately aligned in Mauve^48^, along with their closest neighbors (Supplementary File 1). SNP coordinates were isolated and characterized in Artemis^49^.

### Genomic diversity and dynamics

The genomic diversity was determined based on the strains’ accessory genomes. For each OG a binary measure of presence (1) or absence (0) of a representative protein from a given strain was generated. The contribution of each strain to an OG was therefore denoted by a single numeric value (1 or 0) to represent the presence or absence of a gene, or a group of paralogs in a certain strain. This information was collated into a gene presence/absence matrix (Supplementary File 2). Using the method described by Dutilh *et al.*^50^, the genomic variations among strains collected within each year were calculated. Core OGs (those constituted of a single gene from each of the analyzed isolates) were excluded from this analysis.

The time frames applied to detect changes in the proportion of strains carrying a certain OG post PCV7 compared to before were 2001 to 2006; pre-vaccine, and 2008 to 2011; post-vaccine respectively.

### Statistical analysis

The difference in phylogenetic distance among strains collected between 2001 to 2006 and 2007 was tested by an unpaired t-test. Differences in pre- and post-vaccine gene frequencies were tested by Fisher Exact test. For all analyses, the significance level was set at 0.05.

## Additional Information

**How to cite this article**: Cremers, A. J. H. *et al.* The post-vaccine microevolution of invasive *Streptococcus pneumoniae. Sci. Rep.* **5**, 14952; doi: 10.1038/srep14952 (2015).

## Supplementary Material

Supplementary Information

Supplementary Table 1

Supplementary Table 2

Supplementary Table 3
