# Supplementary material for: The post-vaccine microevolution of invasive Streptococcus pneumoniae
Source: Sci Rep. 2015 Oct 23;5:14952. doi: 10.1038/srep14952 (PMC4615977; doi:10.1038/srep14952)
Supplement: Supplementary Information [file srep14952-s1.docx]

Supplementary Information

**The post-vaccine microevolution of invasive *Streptococcus pneumoniae***

Amelieke J.H. Cremers^1,+^, Fredrick M. Mobegi^1,2,+^, Marien I. de Jonge^1^, Sacha A.F.T. van Hijum^2^, Jacques F. Meis^3,4^, Peter W.M. Hermans^1,‡^, Gerben Ferwerda^1^, Stephen D. Bentley^5^, Aldert L. Zomer^1,2,^*

*^1^Radboud university medical center, Laboratory of Pediatric Infectious Diseases, Nijmegen, The Netherlands*

*^2^Radboud university medical center, Bacterial Genomics Group; Center for Molecular and Biomolecular Informatics, Nijmegen, The Netherlands*

*^3^Canisius-Wilhelmina Hospital, Department of Medical Microbiology and Infectious Diseases, Nijmegen, The Netherlands*

*^4^Radboud university medical center, Department of Medical Microbiology, Nijmegen, The Netherlands*

*^5^Wellcome Trust Sanger Institute, Pathogen Genomics group, Hinxton Cambridge, United Kingdom*

*^+^Authors contributed equally to this work*

*^‡^Current address: Janssen Research and Development, Janssen Pharmaceutical Companies of Johnson & Johnson, 2340 Beerse, Belgium.*

**Corresponding author: Email:* [*a.l.zomer@uu.nl*](mailto:a.l.zomer@uu.nl)

**
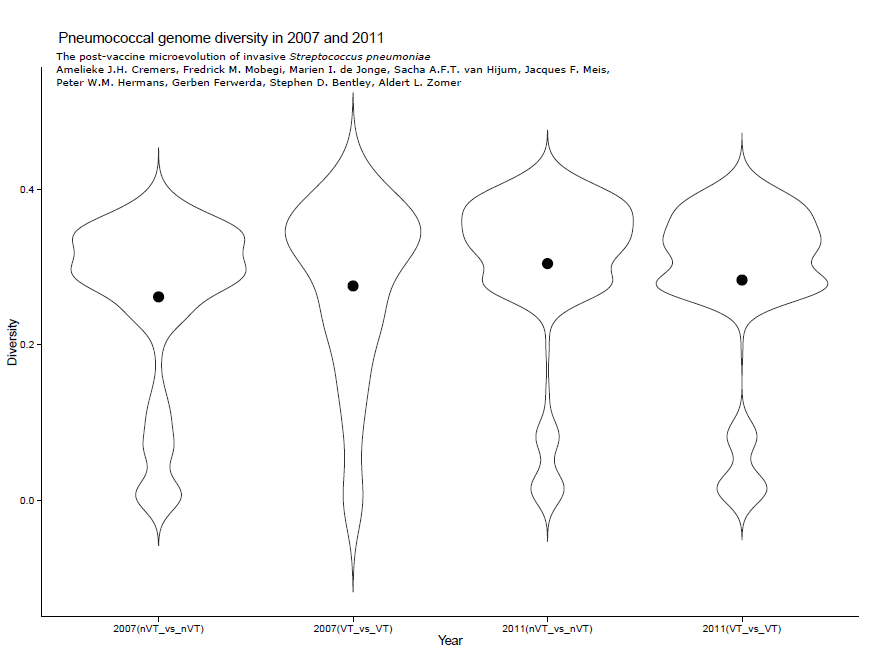
**

**Supplemental Figure 1:** Violin plot showing the post-vaccine accessory genome diversity (middle dot = mean diversity) and population distribution among vaccine serotypes and non-vaccine serotypes. PCV7 was introduced in 2007 and PCV10 in 2011. The plots were generated using the ggplot2 package in R.
